# Radiofrequency Microneedling Versus Fractional Laser for the Treatment of Atrophic Acne Scars: A Systematic Review and Meta‐Analysis

**DOI:** 10.1111/jocd.71025

**Published:** 2026-11-04

**Authors:** Gotof Abdesalam Mustafa, Faisal Fahad AlSuwailem, Fatma H. J. AlAmiri, Rawan Hassan Alzaidi Alotaibi, Zainab Mahmoud Baqer, Leena Abdullah Alotaibi, Sabah Faisal AlQabandi, Reham Alawi Taqi, Suha Giselle Ghanem, Azhar Abbas Ahmed

**Affiliations:** ^1^ Faculty of Medicine University of Khartoum Khartoum Sudan; ^2^ College of Medicine Alexandria University Alexandria Egypt; ^3^ Amiri Hospital Kuwait City Kuwait; ^4^ Taif University Ta'if Saudi Arabia; ^5^ College of Medicine Arabian Gulf University Manama Bahrain; ^6^ East Jeddah General Hospital Jeddah Saudi Arabia; ^7^ Ministry of Health Kuwait City Kuwait; ^8^ Faculty of Medicine Iuliu Hațieganu University of Medicine and Pharmacy Cluj‐Napoca Romania; ^9^ King Faisal Specialist Hospital and Research Center Saudi Arabia

**Keywords:** acne vulgaris, atrophic acne scars, CO_2_ laser, Er:YAG laser, fractional laser, radiofrequency microneedling

## Abstract

**Background:**

Atrophic acne scars are a common and distressing complication of acne vulgaris, with limited consensus on optimal treatment. Radiofrequency microneedling (RFM) and fractional laser are widely used modalities, but their comparative effectiveness remains unclear.

**Aims:**

To compare the efficacy and safety of RFM versus fractional laser in treating atrophic acne scars.

**Methods:**

A systematic review and meta‐analysis were conducted following PRISMA guidelines. Five databases were searched up to February 2026 for randomized and prospective comparative studies. The primary outcome was ECCA score reduction; secondary outcomes included clinical improvement, patient satisfaction, and adverse events.

**Results:**

Eight studies were included. Both RFM and fractional laser demonstrated statistically significant improvements in acne scar severity across multiple outcome measures (*p* < 0.05). Patient satisfaction was consistently high, with up to 95% of patients reporting satisfaction in RFM‐treated groups. Several studies demonstrated comparable clinical improvement between RFM and fractional laser, particularly in terms of dermal remodeling and texture enhancement. In addition, RFM was associated with a favorable safety profile, showing fewer pigment‐related adverse events and shorter downtime compared to fractional laser, while reported side effects in both groups were generally mild and transient.

**Conclusions:**

RFM and fractional laser are effective for atrophic acne scars. RFM may offer similar efficacy with improved tolerability. Further high‐quality studies are needed to confirm these findings.

## 1 Introduction

Atrophic acne scarring is a common and therapeutically challenging sequela of acne vulgaris, persisting despite adequate control of active disease. Its pathophysiology involves inflammation‐induced dermal matrix degradation, abnormal wound healing, and reduced collagen deposition, leading to permanent textural changes [1]. Clinically, scars are classified into ice‐pick, boxcar, and rolling types, each differing in morphology and treatment response [2, 3].

Beyond cosmetic concerns, atrophic scars are strongly associated with psychological distress, reduced self‐confidence, and impaired quality of life, emphasizing the need for effective treatment strategies [4, 5].

Energy‐based devices play a central role in management. Fractional laser systems, both ablative and non‐ablative, utilize fractional photothermolysis to create controlled thermal injury, stimulating collagen remodeling while preserving surrounding tissue [6, 7]. Although effective, their use may be limited by pain, prolonged erythema, downtime, and increased risk of post‐inflammatory hyperpigmentation, particularly in darker skin types [6].

RFM has emerged as an alternative modality. By delivering radiofrequency energy directly into the dermis via microneedles, it induces thermal coagulation and collagen remodeling with minimal epidermal damage [8, 9]. This approach may offer a shorter recovery time and improved safety in higher skin phototypes.

Despite widespread use of both modalities, evidence comparing their efficacy and safety remains limited and heterogeneous. Variability in study design, scar assessment scales, and follow‐up durations complicates direct comparison [10].

Therefore, this systematic review and meta‐analysis aims to compare the efficacy, safety, and patient satisfaction of RFM versus fractional laser in the treatment of atrophic acne scars.

## 2 Materials and Methods

### 2.1 Methods

This systematic review was conducted on February 10, 2026, in accordance with the Preferred Reporting Items for Systematic Reviews and Meta‐Analyses (PRISMA) guidelines [11] and the Cochrane Handbook for Systematic Reviews of Interventions [12]. The protocol was pre‐registered in the International Prospective Register of Systematic Reviews (PROSPERO) under the registration number CRD1322677, available at: https://www.crd.york.ac.uk/PROSPERO/view/CRD420261323180

The aim was to evaluate the effectiveness and safety of RFM compared with Fractional Laser resurfacing for treating facial atrophic acne scars in adults.

### 2.2 Search Strategy

A comprehensive search was conducted across Web of Science, SCOPUS, PubMed, Embase, and the Cochrane Library from inception to identify randomized controlled trials (RCTs) and prospective comparative studies evaluating RFM versus Fractional Laser for atrophic acne scars. Two authors developed the search strategy using MeSH terms. Details on search strategy are provided in File S1.

### 2.3 Inclusion and Exclusion Criteria

Eligibility was defined based on study design, study population, interventions, and outcomes.

#### 2.3.1 Inclusion Criteria

1. RCTs (parallel or split‐face) or prospective comparative studies in English.
2. Adults (≥ 18 years) with facial atrophic acne scars (ice‐pick, boxcar, or rolling).
3. Radiofrequency microneedling (RFM), including fractional or non‐insulated microneedle RF.
4. Fractional laser (ablative lasers such as CO_2_ and Er:YAG, or non‐ablative lasers such as Er:Glass), with ≥ 1‐month follow‐up.
5. The primary outcome is the ECCA score. Secondary outcomes include physician‐reported improvement, patient satisfaction, pain (VAS), and adverse events such as post‐inflammatory hyperpigmentation (PIH) and erythema.

#### 2.3.2 Exclusion Criteria

(1) studies with active acne or non‐acne scars, hypertrophic or keloid scars only, (2) combination therapy, (3) lack of RF–laser comparison, laser‐only comparisons, (4) case reports/series, reviews, or animal studies, and (5) abstract‐only publications or studies with insufficient data.

### 2.4 Study Selection

The identified records (301) were imported into EndNote and Mendeley; 176 remained after duplicate removal. Two reviewers independently screened titles and abstracts using Rayyan [13]. Eight studies underwent independent full‐text screening by four reviewers. Disagreements were resolved by consensus.

### 2.5 Data Extraction

Eligible studies underwent data extraction using a standardized form. Extracted data included study ID, study design and location, participants and scars characteristics, intervention and comparator details, and outcomes. Outcome measures comprised mean change in ECCA scores, scar improvement measures, investigator assessment scales, patient satisfaction, and adverse events. Two reviewers independently extracted the data, with disagreements settled by consensus.

### 2.6 Risk of Bias Assessment

The methodological quality of the included studies was evaluated using validated tools according to the study design. RCTs were assessed using the Cochrane Risk of Bias 2 (RoB 2) tool [14], across five domains: the randomization process, deviations from intended interventions, missing outcome data, measurement of the outcome, and selection of the reported results. Each domain was rated as low risk, some concerns, or high risk, with an overall risk of bias judgment assigned for each study.

Non‐randomized studies were evaluated using the Newcastle–Ottawa Scale (NOS) [15]. This tool evaluates studies based on three domains: selection of participants, comparability of study groups, and outcome assessment. Studies were awarded a maximum of nine stars, with higher scores indicating better methodological quality. Scores of 7–9 indicated low risk, 5–6 moderate risk, and less than 5 high risk of bias.

Two reviewers independently assessed risk of bias, resolving disagreements through discussion and consensus. Results were summarized in tables and figures.

### 2.7 Data Synthesis and Analysis

Data synthesis and statistical analyses were performed using Review Manager (RevMan) [16]. Continuous outcomes were pooled as mean difference (MD) or standardized mean difference (SMD) with 95% confidence intervals (CI), depending on measurement scales. Dichotomous outcomes were analyzed using risk ratios (RR) with 95% CIs. Baseline characteristics were summarized as means ± standard deviations (SDs) for continuous variables and numbers and percentages for categorical variables.

The primary outcome was change in acne scar severity measured by the ECCA scale. Secondary outcomes included investigator‐assessed improvement, adverse events, pain intensity measured using the visual analogue scale (VAS), and patient satisfaction. Investigator‐assessed improvement outcomes reported using different ordinal scales were converted into responder rates for quantitative synthesis. Outcomes not convertible into a common statistical metric due to heterogeneity were summarized narratively.

Heterogeneity among studies was assessed using the Chi‐square (*χ* ^2^) test and the *I* ^2^ statistic. A fixed‐effect model was applied when heterogeneity was low to moderate (*I* ^2^ ≤ 50%), whereas a random‐effects model was used when substantial heterogeneity was present (*I* ^2^ > 50%). Statistical significance was defined as *p* < 0.05.

Outcome variations over time are reported as the means ± SDs, with effect size, *p* < 0.05, and 95% CI used to measure intervention efficacy. Intervention evaluation included efficacy, optimal treatment parameters such as wavelength, frequency, duration, and intensity, long‐term sustainability, adverse events, and participant satisfaction.

## 3 Results

### 3.1 Study Selection

An initial title and abstract screening was applied to 176 studies, and 32 studies were retrieved. The final number of the included trials was eight [17, 18, 19, 20, 21, 22, 23, 24], seven of which were randomized studies—only split face studies were found in their search—and one was a non‐randomized comparative study [23]. The literature selection process was conducted using the PRISMA flow diagram as shown in Figure 1.

**FIGURE 1:**
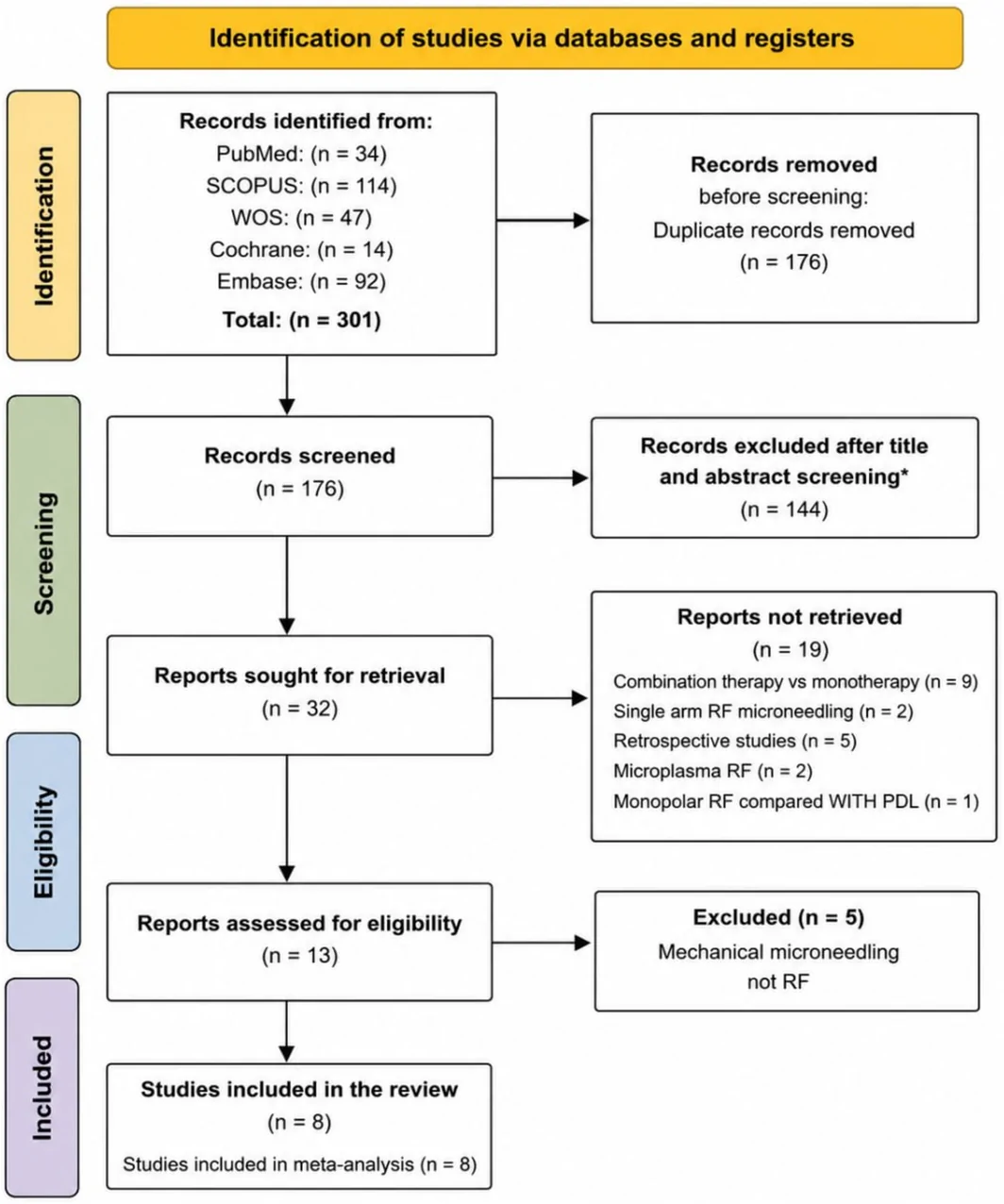
PRISMA flow diagram illustrates the study selection process.

### 3.2 Risk of Bias and Quality Assessment

Two reviewers independently assessed the risk of bias of the included randomized controlled trials using the Cochrane Risk of Bias 2 (RoB 2) tool across five domains: bias arising from the randomization process, bias due to deviations from intended interventions, bias due to missing outcome data, bias in measurement of the outcome, and bias in selection of the reported result.

Four of the seven randomized studies were judged as low risk of bias regarding the overall domain [20, 21, 22, 24]. Kaçar et al. have some concerns related to insufficient reporting of the results and randomization process [17]. Al‐Sultany et al. showed potential issues in the randomization process, suggesting possible selection bias [19]. There are also some concerns in the third and fourth domains. Regarding Al‐Mallah et al., the overall domain was judged as having a high risk of bias due to some concerns in the first, second, and fourth domains [18]. The detailed risk of bias assessment is presented in Figures 2 and 3.

**FIGURE 2:**
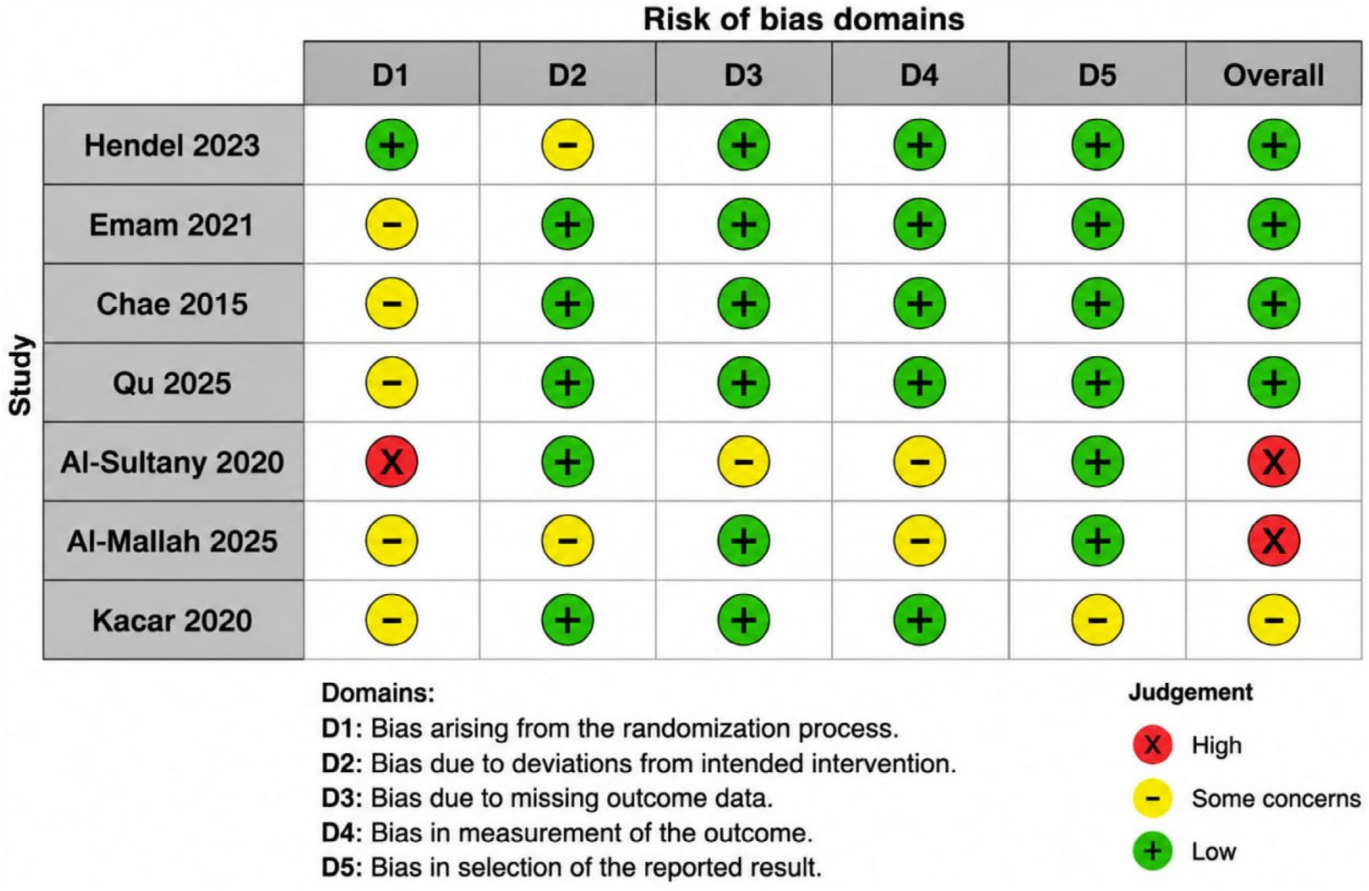
Summary of risk of bias assessment for each included study across the five domains and overall judgment.

**FIGURE 3:**
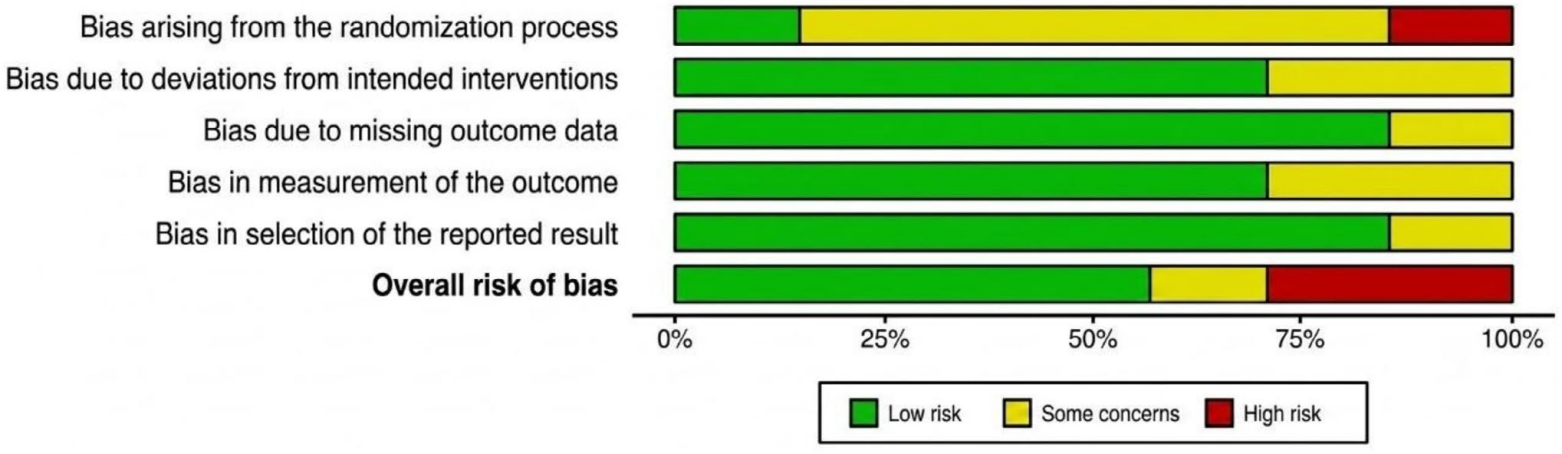
The percentage and summary of risk of bias in the included studies show low risk in the five domains of the three included studies.

In addition, one observational study (Reddy 2021) was assessed using the Newcastle–Ottawa Scale (NOS). The study achieved three, one, and two points in the first, second, and third domains, respectively, for a total score of 6 stars, indicating moderate methodological quality.

### 3.3 Studies Characteristics

Seven of the eight included studies were randomized controlled trials [17, 18, 19, 20, 21, 22, 23, 24] (Table 1). A total of 328 patients were divided into RFM treatment and the fractional laser groups. Interventions included radiofrequency microneedling (RFM) and fractional laser. The treatment parameters for each study were as follows:

**TABLE 1:** Characteristics of the included studies, sociodemographic characteristics of participants, intervention characteristics, comparator characteristics, follow‐up.

Study ID (1st auther, year)	Study design	Sample size (*n*)	Mean age (SD)	Women (%)	Type of Scars RF + Laser	MNRF (intervention)	Fractional Laser (Intervention)	Follow‐up
Al Mallah 2025, Iraq	Open‐label Randomized Controlled Trial	*N* = 30 (RF = 15, Laser = 15)	RF = 24.86 ± 3.22 Laser = 24.86 ± 3.22	RF = 66.7% Laser = 40%	Ice‐pick, Boxcar, Rolling	Microneedling Radiofrequency (MNRF device) 1.5 mm, 30‐61 W, 400‐800 ms, 3 passes, 6 session, 4 weeks	Fractional CO_2_ laser 10 600 nm, pulse 1, 8 ms, 6 session, 4 weeks	1–3 months after final treatment Completion rate 100%
Hendel 2023, Denmark	Randomized split‐face comparative trial (blinded evaluation)	*N* = 30 (RF = 15, Laser = 15)	RF = NR Laser = NR	RF = 13/15 (86.7%) Laser = 13/15 (86.7%)	Rolling (9), Boxcar (4), Ice pick (2)	Genius microneedle radiofrequency 1–3.0 mm, 30‐98mj, 4 passes, 1 session	Ultrapulse deepFX/Active Fx fractional Co2 Laser 10 600 nm, 15–125 mJ, 5%–15% density, 1 session, 3 passes	3 months Completion rate 100%
Kacar 2021, Turkey	Prospective split‐face single‐blinded controlled clinical study	*N* = 54 (RF = 27, Laser = 27)	RF = 23.9 ± 4.4 Laser = 23.9 ± 4.4	RF = 70.4% Laser = 70.4%	Boxcar, rolling, ice‐pick, mixed	FraxisDuo (FRF) 1.5‐3 mm, 30%–50% intensity, 140–160 ms, single pass, 3 session, 1 month	Fractional CO_2_ laser (fco2) 10 600 nm, 12–15 mJ, 256 dots, 1 pass, 4 session, 1 month	1, 2, 3, 6 months Completion rate 100%
Reddy 2021, India	Prospective comparative clinical study (two parallel groups)	*N* = 30 (RF = 15, Laser = 15)	RF = 25.86 years (mean) Laser = 27.40 years	RF = NR Laser = NR	Atrophic acne scars	Microneedling Fractional Radiofrequency (MR 16‐2SB) 0.5–3.5 mm, 50 W, 300 ms, 2 passes, 4 session, 4 weeks	Fractional CO_2_ laser (RF30) 10 600 nm,10–30 mJ, 1, 1 mm, 2 passes, 4 session, 4 weeks	1 month after last session Completion rate 100%
Al‐Sultany 2020, Iraq	Randomized control trial	*N* = 42 (RF = 21, Laser = 21)	RF = 36 Laser = 36	RF = 71.50% Laser = 71.50%	Atrophic (ice‐pick/boxcar/rolling)	Microneedling Fractional RF 0.8–1.2 mm, 50 W, 200–400 ms, 4 sessions, 4 weeks	Fractional CO_2_ laser 10 600 nm,15–25 mJ, 100MTZ/cm2, 4 session 1 month	6 months Completion rate 100%
Chae 2015, South Korea	Randomized controlled trial	*N* = 40 (RF = 20, Laser = 20)	RF = 28.3 ± 5.39 Laser = 25.5 ± 3.76	RF = 20% Laser = 35%	Atrophic (ice‐pick/boxcar/rolling)	Inskin RF microneedling 2 mm, 40–60 W0, 1 ms, 2 passes, 3 sessions, 4 weeks	Sellas 1550 nm, 15–20 mJ/MTZ, 500MTZ/cm 2, 2 passes, 3 session,4 weeks	20 weeks Completion rate 100%
Qu 2025, China	Randomized split‐face clinical study	*N* = 60 (RF = 30, Laser = 30)	RF = 26.9 ± 5.7 Laser = 26.9 ± 5.7	RF = 53.30% Laser = 53.30%	Atrophic (ice‐pick/boxcar/rolling)	Intensif 1.5–2.5, 12–17 W, 80–110 ms,2–3 passes, 1 session	Ultrapulse CO_2_ laser 10 600 nm, 10–12.5 mJ, 5% density, 1 session, 1 pass	1, 2, 3, 6 months Completion rate 100%
Emam 2021, Egypt	Randomized split‐face clinical trial	*N* = 42 (RF = 21, Laser = 21)	RF = 27.29 ± 6.10 Laser = 27.29 ± 6.10	RF = 57.10% Laser = 57.10%	Atrophic acne scars (boxcar, ice‐pick, rolling)	Vivace microneedling RF 2.5 mm,2 MHz, 600 ms, 2 passes, 4 session, 4 weeks	Fontona Xs Dynamis 2940 nm, MSP (100 μs) + SP (300 μs) + XLP (1500 μs), 3 passes, 4 sessions, 4 weeks	3 months after last session Completion rate 100%

Kaçar: FraxisDuo (*n* = 27, energy level 30%–50% intensity) versus fractional laser (FCO_2_) (*n* = 27, wavelength 10 600) [17].

Al Mallah: MRNF (*n* = 15, energy level 30–61 W) versus Fractional CO_2_ laser (*N* = 15, wavelength 10600 nm) [18].

Al Sultany: Microneedling Fractional RF (*n* = 21, energy level 50 W) versus Fractional CO_2_ Laser (*n* = 21, wavelength 10 600 nm) [19].

Qu: Intensive RF (*n* = 30, energy level 12–17 W) versus Ultrapulse CO_2_ laser (*n* = 30, wavelength 10600 nm) [20].

Chae: In skin RF microneedling (*n* = 20, energy level 40–60 W) versus Sellas laser (*n* = 20, wavelength 1550 nm) [21].

Emam: Vivace RF microneedling (*n* = 21, energy level 2 MHz) versus Fotona Xs Dynamis (*n* = 21, wavelength 2940 nm) [22].

Reddy: MR 16‐2SB (*n* = 15, energy level 50 W) versus fractional CO_2_ laser (RF30) (*n* = 15, wavelength 10 600 nm) [23].

Hendel: Genius microneedle radiofrequency (*n* = 15, energy level 30–98mj) versus Ultrapulse deep FX/Active CO_2_ (*n* = 15, wavelength 10 600 nm) [24].

### 3.4 Sociodemographic Characteristic

The studies analyzed from the pooled analysis for RFM were 149 subjects across the seven study arms that demonstrated a mean age of 25.57 years (SD = 6.43), as well as for fractional laser subgroups of 149 subjects had a mean age of 27.49 years (SD = 6.21) (Table 2). There was no statistically significant difference between groups, where MD showed −0.09 years with 95% CI (−1.17 to 0.99; *p* = 0.87), with no heterogeneity (*I* ^2^ = 0%), indicating a fairly distribution of age between the intervention and compared groups.

**TABLE 2:** Improvement in acne scar severity using scales other than ECCA.

Study	Microneedling radiofrequency	Fractional laser	Assessment tool	Outcome reporting	Key findings
Al‐Mallah et al.	57.2% reduction in mean acne‐scar score	59.31% reduction in mean acne‐scar score	Goodman and Baron Global Quantitative Acne Scar Grading System (GBGQASGS)	Pre‐ and post‐treatment mean scores with percentage reduction	Both modalities showed marked scar‐severity reduction
Reddy et al.	3‐grade reduction: 3/15 (20.0%); 2‐grade reduction: 10/15 (66.6%); very good improvement: 3/15 (20.0%)	3‐grade reduction: 2/15 (13.3%); 2‐grade reduction: 9/15 (60.0%); very good improvement: 2/15 (13.3%)	Goodman and Baron qualitative and quantitative grading systems	Categorical grade reduction and quantitative improvement after treatment	Improvement was documented in both groups, with slightly higher response frequencies in the MNRF group
Hendel et al.	Median 1‐point texture improvement at 3 months; best response reached 3‐point improvement	Median 1‐point texture improvement at 3 months; best response reached 3‐point improvement	Texture improvement scale (0–10 visual descriptive scale)	Descriptive evaluation of global skin texture after a split‐face treatment	Best responses reached a 3‐point improvement in 3 treatment areas (10%)

Scar severity was appreciated among six studies. The pooled mean severity score was 55.11 (SD = 43.21) in the MNRF group and 54.94 (SD = 41.42) in the fractional laser group (*n* = 134). Using the Goodman and Baron scale and ECCA score, no statistically significant difference was observed between the two treatment groups. The SD assured −0.00 with 95% CI (−0.24 to 0.24, *p* = 0.99), with no heterogeneity (*I* ^2^ = 0%).

Analysis restricted to studies using ECCA scores also showed no significant difference between MNRF and fractional laser groups showing MD = 2.00 with 95% CI (−2.68 to 6.68, *p* = 0.40), with no heterogeneity (*I* ^2^ = 0%), confirming consistency across studies.

### 3.5 ECCA Score Reduction

The ECCA score is a quantitative clinical scoring system used to assess the severity of atrophic acne scars. Three studies [17, 20, 21] were included in the meta‐analysis, as they exclusively reported acne scar severity reduction using the ECCA scale. This outcome was considered the primary outcome of the study. Pooled analysis demonstrated no statistically significant difference between fractional laser and RFM in the ECCA score reduction (MD = −0.35; 95% CI: −4.53 to 3.83; *p* = 0.87). Moderate heterogeneity was observed among the included studies (*I* ^2^ = 45%, *p* = 0.16), while overall results remained consistent. As shown in Figure 4.

**FIGURE 4:**
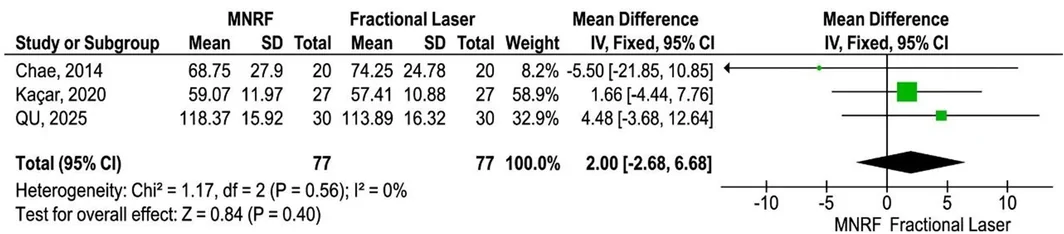
Forrest Plot comparing the ECCA score reduction values between MNRF and Fractional laser.

### 3.6 Change in Acne Scar Severity (Improvement)

In Al‐Mallah et al. [18] scar severity was assessed using the Goodman and Baron Global Quantitative Acne Scar Grading System (GBGQASGS). The mean acne‐scar score decreased by 57.2% in the MNRF group and 59.31% in the fractional laser group.

In Reddy et al. [23] both qualitative and quantitative Goodman and Baron scales were applied. One month after the fourth session, 3/15 patients (20.0%) in the MNRF group showed a 3‐grade reduction, compared with 2/15 patients (13.3%) in the fractional CO_2_ laser group. A 2‐grade reduction was observed in 10/15 patients (66.6%) in the MNRF group versus 9/15 patients (60.0%) in the fractional CO_2_ laser group. Quantitatively, very good improvement was reported in 3/15 MNRF‐treated patients (20.0%) and in 2/15 fractional CO_2_‐treated patients (13.3%).

In Hendel et al. [24] acne‐scar texture was evaluated on a 0–10 visual scale following a single split‐face treatment. At 3 months, both ablative fractional CO_2_ laser and MNRF produced a median 1‐point texture improvement, with a maximum 3‐point improvement observed in three treatment areas (10%).

### 3.7 Subject Satisfaction

Patient satisfaction was reported using different scales and at different follow‐up times (Table 3).

**TABLE 3:** Subject satisfaction.

Study ID	Assessment method	MNRF	Fractional laser	Time point
Qu 2025	4‐grade satisfaction scale	95% satisfied/very satisfied	Not clearly separated by treatment arm in sheet	1, 2, 3, 6 months
Reddy 2021	Patient satisfaction VAS (0–4)	Mean score 2.1	Not clearly reported separately in sheet	1 month after last session
Hendel 2023	Patient satisfaction VAS (0–10)	Median 6 (range 4–10)	Not reported separately in sheet	3 months follow‐up
Emam 2021	Likert satisfaction scale (1–5)	42.9% very satisfied, 38.1% satisfied, 19% neutral	Laser results also reported but percentages combined in sheet	3 months after last session
Chae 2015	5‐point patient satisfaction scale	Patient satisfaction reported	Patient satisfaction reported	20 weeks
Kaçar 2021	VAS satisfaction scale	Scale used but detailed results unclear in extraction sheet	—	Multiple follow‐up visits

Qu et al. [20] used a four‐grade satisfaction scale, reporting that 95% of patients were satisfied or very satisfied, although group‐specific values for MNRF and fractional laser were not provided.

Emam et al. [22] applied a 5‐point Likert scale, where 42.9% of MNRF‐treated patients were very satisfied, 38.1% satisfied, and 19.0% neutral at 3 months post‐treatment.

Reddy et al. [23] assessed satisfaction using a 0–4 visual analogue scale, with a mean score of 2.1 in the MNRF group at 1 month after the final session.

Hendel et al. [24] used a 0–10 visual analogue scale, with a median satisfaction score of 6 (ranges 4–10) at 3 months. Chae et al. [21] assessed satisfaction with a 5‐point scale at 20 weeks, and Kaçar et al. [17] applied a visual analogue scale at multiple follow‐up visits; however, precise group‐specific values were not extractable.

### 3.8 Adverse Events

The most frequently reported adverse events included post‐inflammatory hyperpigmentation, erythema, edema, and treatment‐related pain. Post‐inflammatory hyperpigmentation occurred significantly less frequently with microneedle radiofrequency compared with fractional laser therapy (RR = 0.21, 95% CI: 0.08–0.52; *p* = 0.0009), with no heterogeneity (*I* ^2^ = 0%) (Figure 5).

**FIGURE 5:**
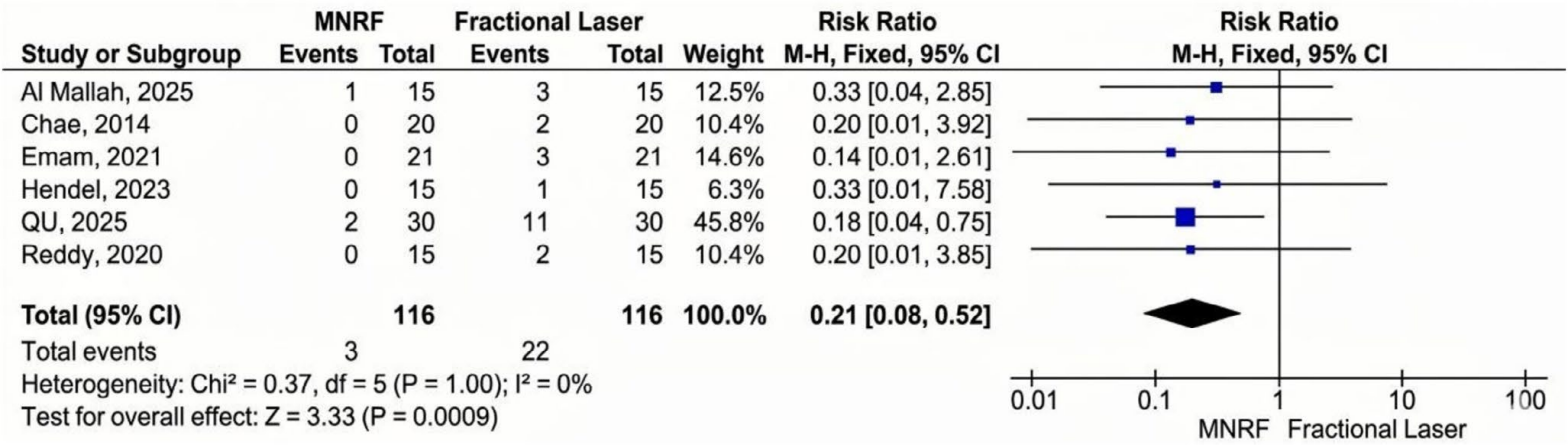
Hyperpigmentation event in MNRF and Fractional laser.

No statistically significant differences were observed between the two modalities for erythema (RR = 0.99, 95% CI: 0.88–1.12; *p* = 0.90) (Figure 6) or edema (RR = 0.96, 95% CI: 0.87–1.06; *p* = 0.40) (Figure 7).

**FIGURE 6:**
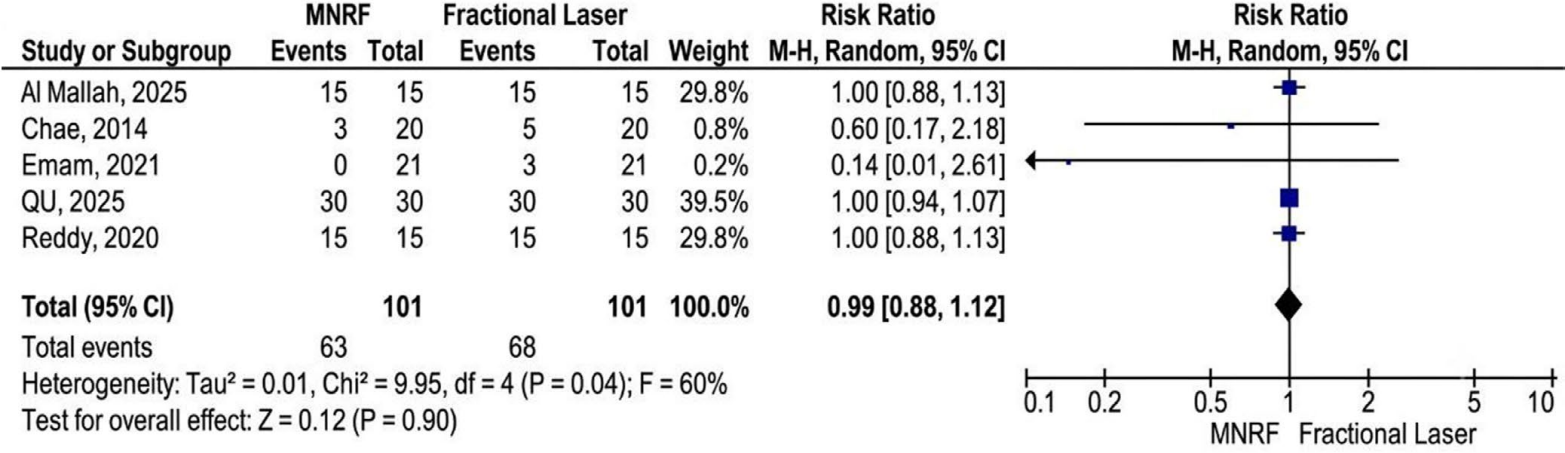
Erythema event in MNRF and Fractional laser.

**FIGURE 7:**
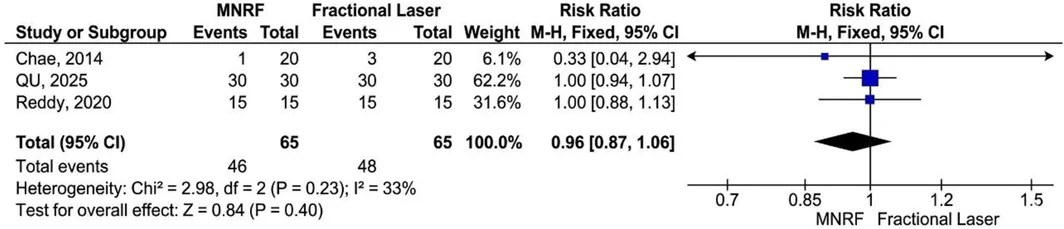
Edema event in MNRF and Fractional laser.

Pain assessed using VAS scores also showed no significant difference between treatments (SMD = −0.46, 95% CI: −2.61 to 1.70; *p* = 0.68) (Figure 8).

**FIGURE 8:**
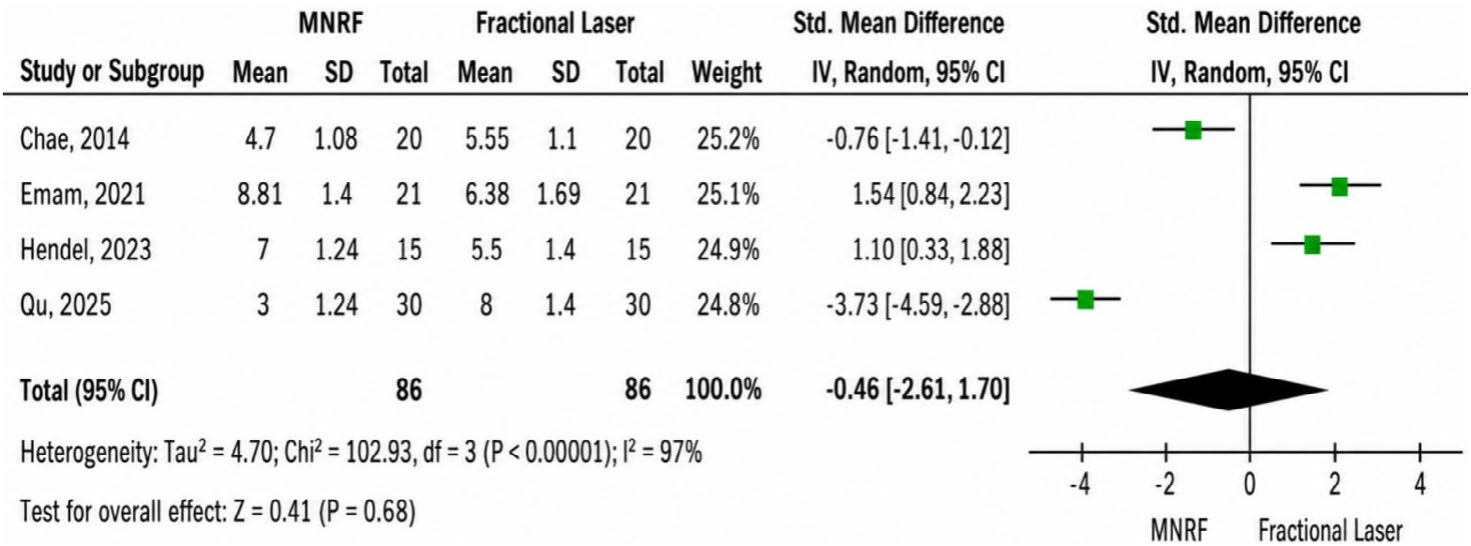
VAS pain score in MNRF and Fractional laser.

## 4 Discussion

This study compared the efficacy and safety of microneedling and fractional laser in the treatment of atrophic acne scars. The results showed that both ablative fractional laser and RFM were effective, with favorable safety profiles.

All studies revealed that both treatments resulted in significant improvement in scar severity using different scores such as the Échelle d'Évaluation Clinique des Cicatrices d'Acné and Goodman and Baron scales [17, 18, 19, 20, 21, 22, 23, 24]. These findings are consistent with previous reviews supporting the effectiveness of these modalities in managing acne scars. Fractional laser has been associated with better skin smoothness and overall improvement in lesion scores [25], while microneedling has shown significant objective scar improvement [26].

The findings of this meta‐analysis—demonstrating that Radiofrequency Microneedling (RFM) and Fractional Laser (FCL) provide comparable efficacy in ECCA score reduction (*p* = 0.87)—are strongly supported by the systematic review conducted by Xiang et al. (2026). By synthesizing 21 studies, Xiang et al. confirmed that most clinical evidence does not report a statistically significant difference in total efficacy between the two modalities [27].

Clinical improvement was noted after one or two sessions and maintained during follow‐up.

For example, Kaçar et al. reported that more than 90% of patients reported improvement in scar severity with both treatments after the first session, with progressive improvement over subsequent sessions [17].

Some studies suggested slightly greater efficacy with fractional laser compared to RFM [17, 18]. Certain studies suggested better responses among female patients, particularly with RFM [17, 19]. Interestingly, combining both treatment modalities appeared to produce the most pronounced improvement in scar scores [17, 18].

In terms of safety, both treatments were generally well tolerated, with mild and transient adverse effects. There were no serious complications. The most common side effects were post‐inflammatory hyperpigmentation followed by local erythema, edema, and pain.

Several studies reported fewer adverse events with RFM compared to fractional laser [20, 22, 23]. Laser‐treated areas were more likely to experience prolonged erythema, swelling, and, in some cases, scarring [17, 18, 20]. In addition, post‐inflammatory hyperpigmentation was more frequently reported with fractional laser, whereas microneedling RF was associated with fewer pigmentary changes and a shorter recovery period. Similar findings were also reported [28, 29].

These differences are explained by their different mechanisms of fractional laser induces thermal injury through ablative columns, stimulating collagen remodeling but increasing the surface damage and recovery time. In contrast, RFM delivers energy directly into the dermis while sparing the epidermis, leading to collagen stimulation with less surface damage [30]. Surprisingly, some studies reported higher perceived pain with RFM despite milder visible reactions [24].

Patient satisfaction was evaluated using visual analogue scale scores. While some studies reported higher satisfaction rates with RFM treatment [23, 24], others showed no differences between treatments [17].

Although fractional laser showed better results than microneedling, the choice of treatment should be tailored to each patient's needs and preferences. RFM might be the better choice for patients at high risk of post‐inflammatory hyperpigmentation or those who want a quicker recovery. On the other hand, fractional laser could be suitable for individuals seeking more effective results. The correlation between objective scar improvements and subjective patient satisfaction supports the clinical effectiveness of both modalities. Pain assessment across studies may have been influenced by the use of topical or local anesthesia, which was inconsistently reported. This variability limits the comparability of VAS pain scores between treatment modalities.

## 5 Conclusion

In conclusion, ablative fractional laser and microneedling fractional radiofrequency are both effective and safe treatments for atrophic acne scars. They show significant and quick improvement, with high patient satisfaction. Microneedling fractional radiofrequency has lower rates of side effects and a shorter recovery time. On the other hand, fractional laser may provide greater effectiveness. It is important to choose the right treatment based on the patient's characteristics, the risk of post‐inflammatory hyperpigmentation, and the treatment goals. More large‐scale randomized controlled trials are needed.

## Author Contributions

G.A.M. contributed to manuscript writing, full‐text screening, and data extraction. F.F.A. and F.H.J.A. contributed to full‐text screening and data extraction. L.A.A. contributed to title and abstract screening, full‐text screening, and manuscript writing. R.H.A.A. contributed to title and abstract screening, full‐text screening, and risk of bias assessment. Z.M.B. contributed to manuscript writing and risk of bias assessment. S.F.A. and R.A.T. contributed to manuscript writing. S.G.G. contributed to database searching and journal submission. A.A.A. contributed to supervision and critical revision of the manuscript. All authors contributed to study selection, critically revised the manuscript, and approved the final version.

## Ethics Statement

This study is a systematic review of previously published studies and did not involve direct patient participation; therefore, formal ethical approval was not required.

## Consent

The authors have nothing to report.

## Conflicts of Interest

The authors declare no conflicts of interest.

## Supporting information


**File S1:** Full Search Strategy.

## Data Availability

Data sharing not applicable to this article as no datasets were generated or analysed during the current study.
