# Supplementary material for: Radiofrequency Microneedling Versus Fractional Laser for the Treatment of Atrophic Acne Scars: A Systematic Review and Meta‐Analysis
Source: J Cosmet Dermatol. 2026 Nov 4;25(11):e71025. doi: 10.1111/jocd.71025 (PMC13736136; doi:10.1111/jocd.71025)
Supplement: Supplementary file 1 [file JOCD-25-e71025-s001.docx]

**Supplementary File 1 - Full Search Strategy**

This systematic review was performed in accordance with the Preferred Reporting Items for Systematic Reviews and Meta-Analyses (PRISMA) guidelines and followed the guidelines provided by the Cochrane Handbook for Systematic Reviews of Interventions.

Systematic retrieval was performed via five electronic databases:

| **Data base** | **Results** |
| --- | --- |
| Pubmed | 34 |
| Embase | 92 |
| Scopus | 114 |
| Cochrane | 14 |
| WOS | 47 |
| Total | 301 |
| Total After duplicates removal: | 176 |

Search Date: February 10, 2026.

Time Frame: From the inception of each database to the date each search string was deployed. The last date of search was: 25/2/2026.

Language restriction: English only.

Study types: Randomized Controlled Trials (RCTs) and prospective comparative clinical trials.

**Primary Search Objective**

To identify studies comparing RF Microneedling effect versus Fractional Laser in treating atrophic acne scars in terms of safety and efficacy.

**Search Strategy Used Across Five Electronic Databases**

**MeSH terms**

| **Population** | **Intervention** | **Comparison** |
| --- | --- | --- |
| "Acne Vulgaris"[MeSH] | ‘’ Radiofrequency Microneedling"[MeSH] | **"**Carbon Dioxide Lasers"[MeSH] |
| "Cicatrix"[MeSH] | "Radiofrequency Therapy"[MeSH] | "Erbium"[MeSH] |
| “Scar”[MeSH] | "Microneedling"[MeSH] | ’Fractional Laser’’ [MeSH] |
| ‘’ post-acne scars’’ [MeSH] | ‘’RF microneedling"[MeSH] | ‘’ fractional CO2’’ [MeSH] |
| atrophic acne scars [MeSH] | ‘’fractional microneedle RF"[MeSH] | "Laser Therapy"[MeSH] |
|  | ‘’NIMFRF "[MeSH**]** | "Erbium:YAG Lasers"[MeSH] |
|  |  | ’non-ablative fractional laser"[MeSH] |
|  |  | ‘’Er:Glass"[MeSH] |
|  |  | ‘’Er:YAG"[MeSH] |

**Query;**

("Acne Vulgaris"[MeSH] OR acne scar* OR atrophic acne scar* OR post-acne scar*)

AND

("Radiofrequency Therapy"[MeSH] OR "Microneedling"[MeSH] OR radiofrequency microneedling OR fractional microneedle RF OR NIMFRF)

AND

("Laser Therapy"[MeSH] OR "Carbon Dioxide Lasers"[MeSH] OR "Erbium:YAG Lasers"[MeSH] OR fractional laser OR CO2 laser OR Er:Glass)

AND

("Randomized Controlled Trial"[Publication Type] OR randomized OR split-face OR comparative study)

**Screening and Selection Process**

Two reviewers independently screened the titles and abstracts for relevance via Rayyan AI. The remaining reports underwent independent full-text screening to confirm compliance with the eligibility criteria. Conflicts were resolved through discussion.
